# Editorial note: possible undisclosed conflict of interest

**DOI:** 10.2807/1560-7917.ES.2020.25.30.2007301

**Published:** 2020-07-30

**Authors:** Eurosurveillance editorial team

**Affiliations:** 1 European Centre for Disease Prevention and Control (ECDC), Stockholm, Sweden

On 10 June 2020, the editorial team was contacted about a perceived conflict of interest for Olfert Landt, CEO of Tib-Molbiol, who was among co-authors of the article Detection of 2019 novel coronavirus (2019-nCoV) by real-time RT-PCR by Corman et al. published in *Eurosurveillance* on 23 January 2020. No conflict was declared for any of the authors under the Conflict of interest section.

At the time of the publication, SARS-CoV-2 had been identified only 16 days earlier as the causing agent of coronavirus disease (COVID-19) and a viral genome sequence had been released on 10 January [1]. Sequences and laboratory protocols developed to detect this novel virus, had been shared by Corman et al. via the World Health Organization (WHO) website already from 13 January onwards and been updated on 17 January [2,3].

The article in *Eurosurveillance* further detailed the protocols for a screening test and its validation. Making the sequences and protocols publicly freely available (alongside other protocols) in a timely manner, and wide distribution of this information through the WHO website and an open access journal, has allowed researchers and laboratories worldwide to rapidly implement virus testing. Detection is crucial for the public health response to any emerging pathogen posing a health threat. The published article describes a generic protocol and does neither refer to primers or probes from a specific company nor does it evaluate a specific kit. Affiliations for all authors were detailed including that of Tib-Molbiol for Olfert Landt.

In response to the concern flagged, we asked all authors to re-confirm their absence of a conflict of interest. We also received a detailed statement from the corresponding author, Christian Drosten, and from Olfert Landt, who explained that for the main reasons outlined above, they do not consider that being Tib-Molbiol’s CEO constituted a conflict of interest with respect to the article in question at the time of submission. They further confirmed that that reagent sets produced and marketed by Tib-Molbiol are different from those in the protocol published in the article and that they were validated independently from this work.

Considering the above, and following consulation with experts in conflict of interest and research integrity and the journal’s associate editors, the *Eurosurveillance* editor-in-chief decided to amend the conflict of interest note by adding the following statement:

*Olfert Landt is CEO of Tib-Molbiol; Marco Kaiser is senior researcher at GenExpress and serves as scientific advisor for Tib-Molbiol.*

This change aims at further enhancing transparency and does not imply a judgment on whether or not a conflict of interest exists.
